# Hybrid rf SQUID qubit based on high kinetic inductance

**DOI:** 10.1038/s41598-018-27154-1

**Published:** 2018-07-03

**Authors:** J. T. Peltonen, P. C. J. J. Coumou, Z. H. Peng, T. M. Klapwijk, J. S. Tsai, O. V. Astafiev

**Affiliations:** 1 RIKEN Center for Emergent Matter Science, Wako, Saitama 351-0198 Japan; 2 Low Temperature Laboratory, Department of Applied Physics, Aalto University School of Science, P.O. Box 13500, FI-00076 Aalto, Finland; 3 Kavli Institute of Nanoscience, Delft University of Technology, Lorentzweg 1, 2628 CJ Delft, The Netherlands; 4 Key Laboratory of Low-Dimensional Quantum Structures and Quantum Control of Ministry of Education, Department of Physics and Synergetic Innovation Center for Quantum Effects and Applications, Hunan Normal University, Changsha, 410081 China; 5 Physics Department, Moscow State Pedagogical University, Moscow, 119435 Russia; 6 Department of Physics, Tokyo University of Science, Kagurazaka, Tokyo 162-8601 Japan; 7 Royal Holloway, University of London, Egham, Surrey TW20 0EX United Kingdom; 8 National Physical Laboratory, Hampton Road, Teddington, TW11 0LW UK; 9 Moscow Institute of Physics and Technology, 141700 Dolgoprudny, Moscow Region Russia

## Abstract

We report development and microwave characterization of rf SQUID (Superconducting QUantum Interference Device) qubits, consisting of an aluminium-based Josephson junction embedded in a superconducting loop patterned from a thin film of TiN with high kinetic inductance. Here we demonstrate that the systems can offer small physical size, high anharmonicity, and small scatter of device parameters. The work constitutes a non-tunable prototype realization of an rf SQUID qubit built on the kinetic inductance of a superconducting nanowire, proposed in Phys. Rev. Lett. 104, 027002 (2010). The hybrid devices can be utilized as tools to shed further light onto the origin of film dissipation and decoherence in phase-slip nanowire qubits, patterned entirely from disordered superconducting films.

## Introduction

Various applications of superconducting quantum bits (qubits), see for example refs^1,2^, benefit from building blocks with good reproducibility of device parameters, high anharmonicity of the energy level spacings, and compact physical size. These requirements apply to superconducting Josephson metamaterials^3–7^, and in particular to the case of superconducting quantum metamaterials^8,9^ where a large number of identical or controllably different “artificial atoms” are required. In typical flux qubits^10^ based on three or four Josephson tunnel junctions (JJs) one of the most significant issues is the exponential sensitivity of the transition frequency on the potential barrier height and hence the precise tunnel junction geometry and transparency. Optimized device design and fabrication process^11,12^ can mitigate this effect along with the steepness of the energy bands and poor decoherence properties away from the optimal flux working point. Promising decoherence times and large anharmonicities have been predicted for inductively shunted JJs^13–15^. They have been realized also experimentally^16–18^, in particular in the fluxonium configuration^16,19,20^, where a single small junction closes a superconducting loop with high inductance, typically formed by a long series array of larger JJs^21,22^.

**Figure 1:**
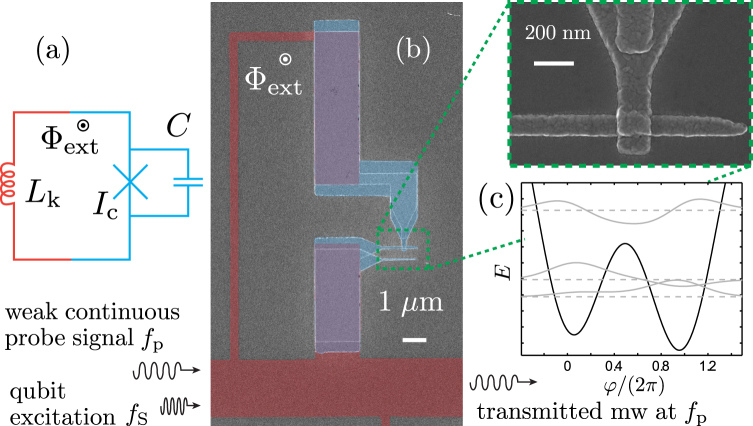
(**a**) Schematic circuit representation of a hybrid rf SQUID. A superconducting loop with high kinetic inductance (red) is closed with a single Josephson junction (blue) and placed into perpendicular external magnetic field. (**b**) False-color scanning electron micrograph of a TiN–Al rf SQUID investigated in this work. The TiN loop is shaded in red, whereas the Al-based tunnel junction is shown in blue, and the direct superconductor-to-superconductor contact overlap areas are colored purple. (**c**) Sketch of the potential *U*(*φ*) (black solid line) for Φ_ext_/Φ_0_ = 0.56, together with the three lowest-lying energy levels (horizontal gray dashed lines) and the corresponding wavefunctions (gray solid lines) from the rf SQUID Hamiltonian for parameters typical to the measured devices.

In this work, we develop and present an experimental study towards flux qubits in the basic rf SQUID geometry of a single Josephson junction shunted by the inductance of a superconducting loop^23–25^. Crucially, in our devices the loop inductance is dominated by the kinetic inductance of a narrow wire patterned from a thin disordered superconducting TiN film, *cf*. Fig. 1. Flux qubit designs combining conventional Al junctions and nanowire kinetic inductors have been first proposed and theoretically analyzed in ref.^13^. Analogously to the use of a long JJ array to form the highly inductive environment for the active qubit junction, this approach allows to realize a large loop inductance in compact size. There are multiple alternatives to TiN as the source of the kinetic inductance. They include nanowires engineered for example out of NbSi^26,27^, MoGe^28^, Ti^29^, granular aluminium^30^, or NbN^13^ and other materials such as NbTiN, WSi, and MoSi studied intensively for superconducting nanowire single photon detectors^31^.

Our motivation for the study of the Al–TiN system is threefold: First, we seek to demonstrate such a hybrid superconducting quantum system, and to investigate the feasibility of this simple archetype of a flux qubit. Secondly, we look to employ the hybrid structure, combining a standard aluminium-based JJ with the loop made of an ultrathin superconductor close to the superconductor-to-insulator transition^32–34^ to provide the high inductance, as a tool to assess film-induced decoherence and dissipation in phase-slip nanowire qubits. Building on the wealth of studies of quantum phase slips in continuous nanowires^28,32,35–39^, structures with a narrow nanowire embedded into a superconducting loop with high kinetic inductance have been proposed^26,40,41^ to be used as flux qubits completely without conventional JJs. Based on coherent quantum phase slips occurring along the nanowire, we have realized such structures patterned in their entirety from InO_x_^42^ and NbN^43^ wires. As a third motivation, our hybrid devices pave the way for transport measurements of phase-slip physics in the basic system of a single JJ and a large inductance^44,45^. This complements earlier experimental works^46,47^ where the inductance is formed by an array of JJs, building on the long-standing study of quantum phase fluctuations in 1D JJ arrays^48–50^.

**Figure 2:**
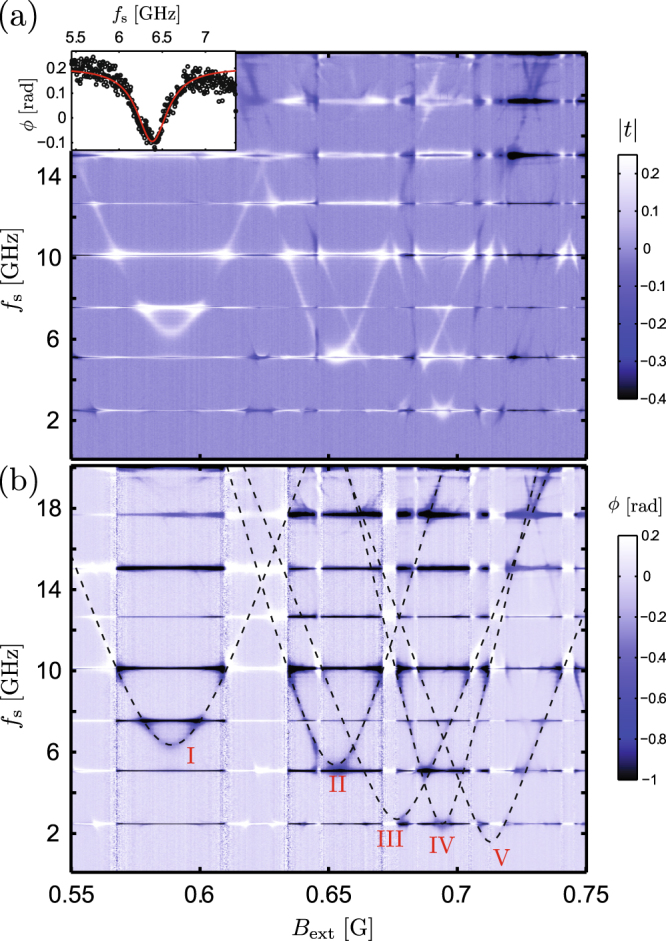
(**a**) Typical two-tone spectroscopy in a narrow range of the external magnetic field *B*_ext_, showing the amplitude change of mw transmission through the resonator, probed at a fixed frequency at one of the resonant modes. The horizontal lines arise due to the multiple resonator modes. Inset: spectroscopy lineshape at the optimal point for the leftmost transition evident in the main panel (device I with Δ/h ≈ 6.3 GHz). (**b**) The same spectroscopy measurement as in panel (a), now showing the phase change of the mw transmission coefficient *t*. The dashed lines correspond to theoretically calculated qubit frequencies *f*_q_ vs. *B*_ext_ for five devices with the strongest signatures in this range of *B*_ext_.

Figure 1(a) shows a schematic of a hybrid rf SQUID of the type described above: The superconducting loop has total kinetic inductance *L*_k_, giving rise to the inductive energy scale ${E}_{{\rm{L}}}={{\rm{\Phi }}}_{0}^{2}\mathrm{/(4}{\pi }^{2}{L}_{{\rm{k}}})$. Here, Φ_0_ = *h*/2*e* denotes the superconducting flux quantum. Likewise, the junction has critical current *I*_c_ and capacitance *C*, resulting in the Josephson energy *E*_J_ = *ħI*_c_/2*e* and charging energy *E*_C_ = *e*^2^/2*C*. The SQUID loop is placed in a perpendicular external magnetic field *B*_ext_, giving rise to the flux Φ_ext_ threading the loop. Figure 1(c) further shows a sketch of the SQUID double well potential *U*(*φ*) = *E*_J_(1 − cos *φ*) + *E*_L_(*φ* − *φ*_ext_)^2^/2 (see, for example, ref.^23^), as well as the three lowest energy levels and wave functions calculated for *φ*_ext_ = 2*π* × 0.56, and the representative parameters *E*_L_/*h* ≈ 4.5 GHz, *E*_J_/*h* ≈ 41 GHz, and *E*_C_/*h* ≈ 18 GHz, yielding a qubit level spacing *f*_q_ ≈ 11.1 GHz between the two lowest levels. These values are close to devices I and II in Fig. 2. Here, the control phase *φ*_ext_ is related to the externally applied biasing magnetic flux Φ_ext_ via *φ*_ext_ = 2*π*Φ_ext_/Φ_0_.

## Sample Details

Fabrication of the hybrid structure is a technologically challenging problem. The key element is a superconducting contact between the thin film of the highly disordered material and Al. The false color scanning electron micrograph in Fig. 1(b) illustrates a typical single rf SQUID studied in this work, together with a sketch of the measurement setup. The approximately 400 nm wide TiN wire that provides the kinetic inductance is shaded in red, whereas the Al-AlOx-Al JJ, fabricated by two-angle shadow evaporation and closing the TiN loop, is highlighted in blue. The two large TiN–Al contact overlap areas are colored purple. The bottom TiN loop edge doubles as part of the 2.5 *μ*m wide resonator center line, widening to 5 *μ*m outside the center section with the SQUID loops. This shared mutual kinetic inductance facilitates the inductive SQUID–resonator coupling.

To pattern inductances from the TiN films, we used a process similar to refs^43,51^, relying strongly on electron beam lithography (EBL). The starting point is an oxidized Si wafer onto which a thin film of TiN with thickness *d* ≈ 6 nm is grown by atomic layer deposition (ALD)^52–54^. This TiN film is identical to film A in ref.^53^. The fabrication process is outlined in the Methods section. A completed SQUID-resonator chip is enclosed in a sample box, and microwave characterization is performed in a dilution refrigerator at the base temperature close to 25 mK. Samples from several fabrication rounds with differing Ar ion cleaning and oxidation parameters were cooled down. Here we present measurement results belonging to one typical sample.

From low temperature dc transport measurements of separate test structures, we infer sufficient quality of the TiN–Al contacts, supporting supercurrents $\gg {I}_{{\rm{c}}}$, the critical current of the SQUID Al junction, and showing no significant suppression of the transition temperature *T*_c_ of the TiN film due to the Ar ion cleaning. Similarly, suitable JJ oxidation parameters were determined by room temperature resistance measurements of a series of junctions with differing overlap areas.

## Microwave Characterization

To characterize the devices we use a vector network analyzer to monitor the transmission of microwaves through the resonator, at probing frequencies *f*_p_ close to one of the resonant modes *f*_*n*_ = *nv*/2*L*, *n* = 1, 2, 3, …. Here, *L* denotes the resonator length and *v* = 1/(*L*_*l*_*C*_*l*_)^1/2^ the effective speed of light, expressed in terms of *L*_*l*_ (*C*_*l*_), the inductance (capacitance) per unit length. The samples reported here contain a resonator with *L* = 1.5 mm, resulting in the fundamental mode frequency *f*_1_ ≈ 2.5 GHz with loaded quality factor *Q*_L_ ≈ 1 × 10^3^.

Signatures from the SQUID loops become visible as the global external magnetic field *B*_ext_ is scanned. In a typical initial test this is done over a period corresponding to Φ_ext_ of several flux quanta through the loops. At the input port of the resonator, the low-power probing tone at frequency *f*_p_ is combined with another continuous microwave signal at frequency *f*_s_ for exciting the qubits. A representative result of such two-tone spectroscopy is illustrated in the top panel of Fig. 2, focused on a range of *B*_ext_ with transitions belonging to five loops coupled to the same resonator. In this measurement, showing the magnitude change of the normalized transmission coefficient *t* relative to a frequency-independent background level, the weak probe tone was fixed at *f*_p_ = *f*_4_ while the frequency *f*_s_ of the strong drive signal was scanned across a large span close to 20 GHz.

The bottom panel of Fig. 2 displays the corresponding phase change of *t*, together with dashed lines indicating qubit transition frequencies calculated according to the standard rf SQUID Hamiltonian^23^

1 $$H={E}_{{\rm{C}}}{\hat{n}}^{2}-{E}_{{\rm{J}}}\,\cos \,\hat{\phi }+{E}_{{\rm{L}}}{(\hat{\phi }-{\phi }_{{\rm{ext}}})}^{2}\mathrm{/2.}$$

They are obtained by finding the lowest energy eigenstates by exact diagonalization. In Eq. (1), the number operator $\hat{n}$ of the charge on the junction capacitor and the phase operator $\hat{\phi }$ obey the commutation relation $[\hat{\phi },\hat{n}]=i$. Close to Φ_ext_ = (*N* + 1/2)Φ_0_, the shape of the curves is well approximated by $h{f}_{{\rm{q}}}=\sqrt{{\varepsilon }^{2}+{{\rm{\Delta }}}^{2}}$. Here *ε* = 2*I*_p_*δ* Φ with *I*_p_ denotes the persistent current, and we introduced the flux deviation from degeneracy, *δ*Φ = Φ_ext_ − (*N* + 1/2)Φ_0_.

The inset of Fig. 2(a) further shows the spectroscopy signal lineshape for the SQUID with Δ/*h* ≈ 6.3 GHz (device I in the main plot), in the low power limit of the spectroscopy tone, together with a Lorentzian fit. For different devices, we find typical HWHM values between 50–300 MHz at the optimal point, depending on the detuning from the nearest resonator modes and transitions due to the other SQUID loops. We emphasize that this is the first study of the hybrid TiN–Al devices, and the coherence can likely be improved by optimizing the geometry and improving the film quality as suggested also by our earlier study of qubits patterned entirely from ultrathin disordered NbN films^51^.

**Figure 3:**
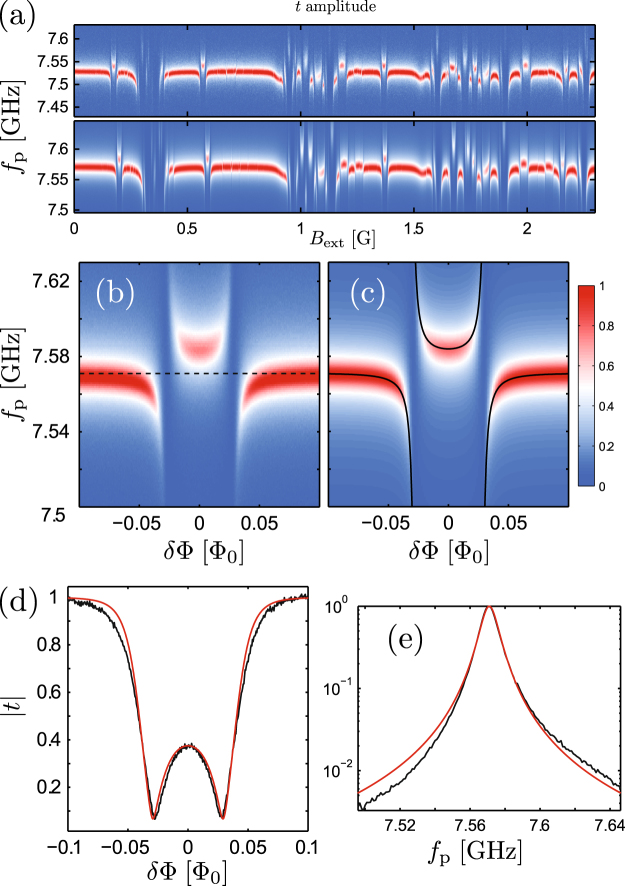
(**a**) Normalized mw transmission coefficient amplitude |*t*| for two nominally identical samples, fabricated simultaneously and characterized in the same cooldown cycle. After detailed analysis, fingerprints from 23 out of the 30 SQUID loops can be distinguished. (**b**) Measured features in |*t*| due to a single rf SQUID, compared to the calculated transmission in (**c**). Panel (d) shows a comparison of line cuts of (**b** and **c**) at constant *f*_p_ = *f*_3_, indicated by the horizontal dashed line in (**b**). In panel (e), the lineshape of the bare resonator mode (black) is compared with a Lorentzian fit (red).

**Figure 4:**
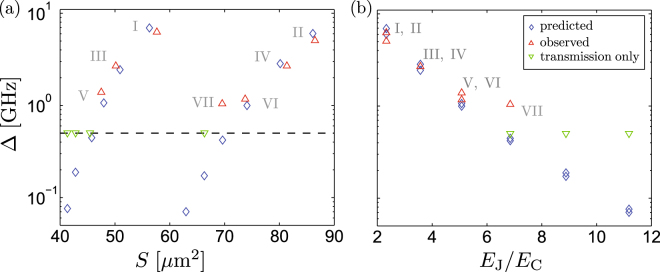
(**a**) Observed rf SQUID energy gaps Δ at the optimal points (*ε* = 0) for one sample. The values of Δ are extracted from two-tone spectroscopy measurements similar to Fig. 2, or indirectly from transmission measurements such as the ones in Fig. 3. They are plotted against an effective loop area obtained from the observed periodicities with magnetic field. The symbols ∇ and Δ show the experimental points. They are compared with theoretical predictions (◇) based on the standard rf SQUID Hamiltonian (see text for details). (**b**) Same as panel (a), plotted as a function of the ratio *E*_J_/*E*_C_.

Figure 3(a) compares the *B*_ext_-dependent transmission amplitudes for *f*_p_ around a narrow range centered at *f*_3_. The two panels correspond to two nominally identical samples cooled down simultaneously, demonstrating good reliability of the TiN–Al contacts and a promising degree of reproducibility. After detailed analysis of the periodicities of the various features, we detect fingerprints from 23 out of the total 30 SQUID loops, with the largest predicted values of Δ. The remaining devices with ${\rm{\Delta }}/h\ll 500\,{\rm{MHz}}$ are likely to be functional as well, although with too weak coupling for their features to be resolved in this measurement. The bottom panel corresponds to the sample in Fig. 2 as well as Fig. 4 below.

The behavior of |*t*| at the individual anticrossings due to the qubit transitions can be modeled accurately using a model based on a standard Lindblad master equation^43,55^. In panel (b) of Fig. 3 we show in an enlarged view the measured features in the normalized transmission amplitude |*t*| due to the anticrossings of a single qubit (device I in Fig. 2). The plot is a zoom-in to a short section of the data in the bottom panel of Fig. 3(a). Panel 3(c) displays the transmission amplitude calculated with the master equation-based model^43,55^, in good agreement with the measurement.

The black solid lines indicate the *B*_ext_-dependence of two of the eigenstates of the hybridized qubit–resonator system. The horizontal black dashed line shows the bare resonator frequency *f*_3_, the value of *f*_p_ at which the 1D line cuts of |*t*| in Fig. 3(d) are plotted as a function of *B*_ext_. In panel (e) we further plot the bare resonator transmission for *f*_p_ around *f*_3_ as the black solid line, at a constant *B*_ext_ when all the qubit transitions are well detuned from this resonator mode. The red line is a Lorentzian fit included for reference.

After comparisons [as in Fig. 3(b–d)] of the transmission measurements with the theoretical model for several qubit transitions visible in both of the two resonators presented in Fig. 3(a), we can indirectly approximate the scatter in Δ to be less than 5% for the qubits with the largest Δ. For the initial samples reported here all the SQUIDs had different parameters by design, mainly the combination of the loop length and junction size. In addition, the number of well-isolated features is limited due to the large number of loops in each resonator. To get a more accurate estimate of the fabrication scatter in Δ and other device properties, future experiments need therefore investigate fewer nominally identical SQUIDs coupled to the same resonator, and include a detailed comparison of two nominally identical resonators.

In Fig. 4 we collect together the minimum qubit energy gaps Δ at the optimal flux points, for one of the measured chips. They are shown as the red upward-pointing triangles, extracted from fits to two-tone spectroscopy measurements similar to Fig. 2. Our present scheme is sufficient for resolving qubits in two-tone spectroscopy if ${\rm{\Delta }}/h\gtrsim 1\,{\rm{GHz}}$. Devices with Δ/*h* < 1 GHz remain visible in direct transmission measurements, *cf*. Fig. 3. However, the exact value for Δ in this case can be only indirectly inferred from a comparison of the numerically simulated transmission coefficient with the measurement. For ${\rm{\Delta }}/h\ll {f}_{{\rm{p}}}$ this leads to a large uncertainty, and hence these devices with low Δ are indicated at 500 MHz (green down-triangles).

Panel (a) of Fig. 4 plots the experimental values of Δ against the effective loop area *S*, deduced from the *B*_ext_ -periodicity of the spectroscopy lines. Analogously, for the SQUIDs with the lowest Δ, the values of *S* were determined by the *B*_ext_ -periodicity of features in direct transmission measurements. The sawtooth behavior evident in Δ vs. *S* in Fig. 4(a) is due to the designed variation in the JJ width.

To compare the observed energy gaps Δ with theoretical predictions, we use the rf SQUID Hamiltonian of Eq. 1. As input parameters we take the sheet kinetic inductance $L\approx 1.2\,{\rm{nH}}$ determined independently from the resonator properties, as well as the nominal loop areas and the number of squares of TiN in each of loops. In addition, we use JJ overlap areas obtained from SEM observations. They differ from the nominal design overlaps, by approximately constant offsets of 50 nm and 30 nm in the width and the height of the junction, respectively. Then, using as adjustable parameters only the values *C*_0_ ≈ 70 fF/*μ*m^2^ and *I*_0_ ≈ 5.2 *μ*A/*μ*m^2^ of the specific junction capacitance and critical current, respectively, we find reasonable overall agreement between the predictions of the model (blue diamonds) and the experimental observations. Notably, we assume the same values for these oxidation parameters for all the junctions. Panel (b) of Fig. 4 re-plots the data in (a), now shown as a function of the expected ratio *E*_J_/*E*_C_. In Table 1 we further collect together the main model parameters for the experimentally detected qubits.

**Table 1:** Parameters of the experimentally detected qubits in Fig. 4.

Device identifier	I	II	III	IV	V	VI	VII
Designed loop area [*μ*m^2^]	56.3	86.1	50.9	80.2	48.0	74.1	69.6
Observed effective area [*μ*m^2^]	57.6	86.5	50.2	81.4	47.5	73.8	69.6
Δ (calculated) [GHz]	6.9	6.0	2.4	2.8	1.1	1.0	0.4
Δ (observed) [GHz]	6.3	5.1	2.7	2.7	1.4	1.2	1.0
${E}_{{\rm{L}}}={{\rm{\Phi }}}_{0}^{2}\mathrm{/(4}{\pi }^{2}{L}_{{\rm{k}}})\,[{\rm{GHz}}]$	5.1	4.2	3.7	4.5	4.0	3.7	4.0
*E*_J_ = *ħI*_c_/2*e* [GHz]	40.9	40.9	50.7	50.7	60.4	60.4	70.2
*E*_C_ = *e*^2^/2*C* [GHz]	17.6	17.6	14.2	14.2	11.9	11.9	10.2

## Discussion

In summary, we have developed and investigated properties of hybrid rf SQUID qubits relying on the high kinetic inductance of a thin, disordered superconducting film. We find reasonable reproducibility of the device parameters. Future samples will benefit from having only one qubit coupled to a single, hanger-style resonator, several of which can be multiplexed to a single readout transmission line. We note that a somewhat thicker TiN film can be straightforwardly used for forming an equally large loop inductance, in the form of a meander. Then, it is likely that making the contact will be easier as well as the qubits are expected to be subjected to less dissipation. Moreover, the Ar milling step can be further separately optimized.

Due to the robust fabrication process, the hybrid rf SQUIDs can be employed as a characterization tool and to provide a further control check of decoherence in phase-slip qubits, pointing towards film losses. The present work, demonstrating the ability to create good contact between the thin TiN film and subsequently evaporated Al structures, will be further relevant for dc transport measurements dealing with phase-slip physics of Josephson junctions in highly inductive environments.

## Methods

### Sample fabrication

We start from an oxidized Si wafer covered by an ALD-grown film of TiN with thickness *d* ≈ 6 nm. First, a mask for the CPW resonator ground planes [not visible in Fig. 1(b)] as well as coplanar transmission lines for connecting to the microwave measurement circuit is defined by EBL. These structures are consequently metallized in an electron gun evaporator with 5 nm Ti, 70 nm Au, and 10 nm Al on top. After liftoff, another layer of resist is applied by spin coating, and patterned in a second step of EBL to act as an etch mask for the TiN loops and the resonator center line, *i*.*e*., the structures highlighted in red in Fig. 1(b). The pattern is transferred into the TiN film by reactive ion etching (RIE) with CF_4_ plasma.

Following the etching step, the remaining resist is removed, and a new bilayer resist is applied to prepare for the last EBL step for defining the Josephson junction, blue in Fig. 1(b), to close the TiN loop. After development, the mask is loaded into an UHV e-gun evaporator. Crucially, prior to Al deposition the exposed TiN contact surfaces, purple in in Fig. 1(b), are cleaned by a brief *in-situ* Argon ion milling. Immediately after this, the typically 30 nm thick Al electrodes of the JJ are deposited by conventional shadow evaporation at two different tilt angles. The two Al depositions are separated by an *in-situ* oxidation in a 10–90% mixture of O_2_ and Ar to form the AlOx tunnel barrier. To protect the TiN film from oxidation, the samples are stored under nitrogen atmosphere, and cooled down within 1–2 days after removing the protective resist.

### Data availability

The datasets generated and analyzed during the current study are available from the corresponding author on reasonable request.
